# Chemo-free salvage treatment outperforms traditional chemotherapy in advanced lines of relapsed/refractory subcutaneous panniculitis-like T-cell lymphoma

**DOI:** 10.3389/fimmu.2024.1476875

**Published:** 2024-12-09

**Authors:** Chao Chen, Jingjing Yin, Minghui Duan, Wei Wang, Danqing Zhao, Chong Wei, Congwei Jia, Wei Zhang, Daobin Zhou, Yan Zhang

**Affiliations:** ^1^ Department of Hematology, Peking Union Medical College Hospital, Chinese Academy of Medical Sciences and Peking Union Medical College, Beijing, China; ^2^ Department of Hematology, Beijing Hospital, National Center of Gerontology, Institute of Geriatric Medicine, Chinese Academy of Medical Sciences, Beijing, China; ^3^ Department of Pathology, Peking Union Medical College Hospital, Chinese Academy of Medical Sciences and Peking Union Medical College, Beijing, China

**Keywords:** subcutaneous panniculitis-like T-cell lymphoma, chemo-free regimen, chemotherapy, immunotherapy, T-cell lymphoma

## Abstract

**Introduction:**

Subcutaneous panniculitis-like T-cell lymphoma (SPTCL) is a rare subtype of non-Hodgkin lymphoma with a good prognosis, but the optimal treatment for relapsed/refractory (R/R) SPTCL has been rarely discussed.

**Methods:**

This study aims to compare the efficacy of conventional chemotherapy and chemo-free immunomodulatory regimen for R/R SPTCL. We retrospectively reviewed the patients with first relapse or primary refractory SPTCL between September 1997 and October 2020.

**Results:**

A total of 19 patients with R/R SPTCL with a median age of 34 were included. All patient received the first-line chemotherapy-based treatment with a median PFS of 1.8 months. In these patients, 16 received salvage second-line treatment with an ORR of 31.3% and a median TTNT of 3.0 months. 13 of these 16 patients received chemotherapy-based treatment, resulting in a median TTNT of 2.4 months. 2 of these 16 patients received allogeneic hematopoietic stem cell transplantation and achieved long term complete remission (CR). In third-line treatment, 7 patients received chemotherapy-based regimen and 6 received chemo-free regimen such as VRMP (bortezomib, lenadomide and methylprednisolone) regimen and CsA plus IFNα regimen. The median TTNT of chemotherapy and chemo-free group were 3.2 months and not reached, respectively.

**Discussion:**

Chemo-free group had a better TTNT than chemotherapy group (p=0.007). The use of chemotherapy-free regimens for R/R SPTCL appears promising and warrants further validation.

## Introduction

According to the 5th edition of the World Health Organization Classification of hematolymphoid tumours, subcutaneous panniculitis-like T-cell lymphoma (SPTCL) is a rare subtype of non-Hodgkin lymphoma which clinically resembles panniculitis and is characterized by infiltration of skin and subcutaneous adipose tissue by cytotoxic T-cells (1, 2). It is defined as a lymphoma of αβ-T-cell origin, which is cytotoxic T-cell mediated (3). SPTCL is estimated to account for 1–2% of cutaneous lymphoma cases, with most studies comprising case reports or small retrospective series. Several studies have summarized the clinic-pathological features, treatment and clinical outcomes of SPTCL, mainly in European countries (4–7). Compared to other T-cell lymphomas, patients with SPTCL typically present at a younger age, with a higher incidence among females than males, and a greater frequency of laboratory abnormalities. The characteristics of SPTCL vary across countries (5).

Currently, no consensus exists on a standard first-line regimen for SPTCL, with chemotherapy response rates ranging between 50% and 71% (5, 6). In Asian studies (5), the complete remission (CR) rate of chemotherapy was approximately 40%. Corticosteroids and immunosuppressive drugs are alternative options for SPTCL, offering higher response rates but shorter durations of disease control (4, 8).

Low disease control rate and high relapse rates necessitate further investigations into relapse or refractory (R/R) SPTCL patients. The key issue is to choose an optimal treatment regimen. Chemotherapy is a treatment option for R/R T-cell lymphoma, but treatments including gemcitabine, liposomal doxorubicin, and polychemotherapy have yielded unsatisfactory results. Gemcitabine produced an ORR of 48%-68% and a CR rate of 9-20% (9) in T-cell lymphoma. Liposomal doxorubicin is currently used for advanced cutaneous T-cell lymphoma, with an ORR of 41-88% and a progress-free survival of 13 months (10). However, there is insufficient evidence indicating superior outcomes with multi-agent chemotherapy in R/R SPTCL. Conversely, several studies applying non-cytotoxic medications have achieved impressive results. Zinzani et al. reported that bortizomib was well-toleranted and demonstrated significant anti-tumor effects in R/R cutaneous T-cell lymphoma in 2007 (11). Cyclosporin A has showed its efficiency in many cases (4, 8). Although Brentuximab vedotin, Mogamulizumab, and Pralatrexate have been extensively utilized in the treatment of CTCL, there have been no reports of their use in the context of SPTCL.

Compared to other regions, it is notable that Chinese patients have a higher mortality rate (5). Few studies reviewing Chinese SPTCL patients have focused on R/R SPTCL treatment regimens. Here we retrospectively studied R/R SPTCL in China to describe the clinical characteristic of R/R SPTCL and compared the efficacy of chemo-free and chemotherapy.

## Patients and methods

### Patients

The local ethical review boards approved this retrospective cohort study, conducted at Peking Union Medical College Hospital. All patients were informed, and the procedure was performed under the Declaration of Helsinki. Patients who were diagnosed as R/R SPTCL between September 1997 and October 2020 in Peking Union Medical College Hospital were included. The biopsy slides were further reviewed by two independent pathologists to confirm the diagnosis. Their clinical profiles were recorded and followed up extensively.

### Efficacy evaluation

Responses to treatment were classified as complete response (CR), partial response (PR), stable disease (SD) or progression disease (PD), based on the Lugano 2014 criteria (12). Relapse was defined as disease relapse after achieving CR or PR after initial therapy. Being refractory to treatment was referred to disease progression during or within 6 months after the initial therapy.

### Statistical analysis

Overall survival(OS) was calculated from the date of the date of diagnosis until the patient’s death or last follow up without an event. Progression-free survival(PFS) was calculated from the date of the start of treatment until either disease progression or death from any cause. Time to next treatment (TTNT) is the interval from the start of the current treatment to the initiation of the next line of therapy. Duration of response (DOR) is the period from the initial documentation of a complete or partial response to the time of disease progression or death. Survival curves were estimated using the method of Kaplan-Meier and statistical comparison between curves was done by log-rank testing. Univariate Cox regression was used to evaluate whether variables had an impact on survival. All reported p-values are two-sided, and values of p<0.05 were considered significant. Relationships between subgroups were examined by Pearson test or the Fisher exact test, where appropriate. Statistical analyses were performed with SPSS 20.0 software and Graphpad prism 7.0.

## Results

### Baseline clinical characteristics

**Figure 1:**
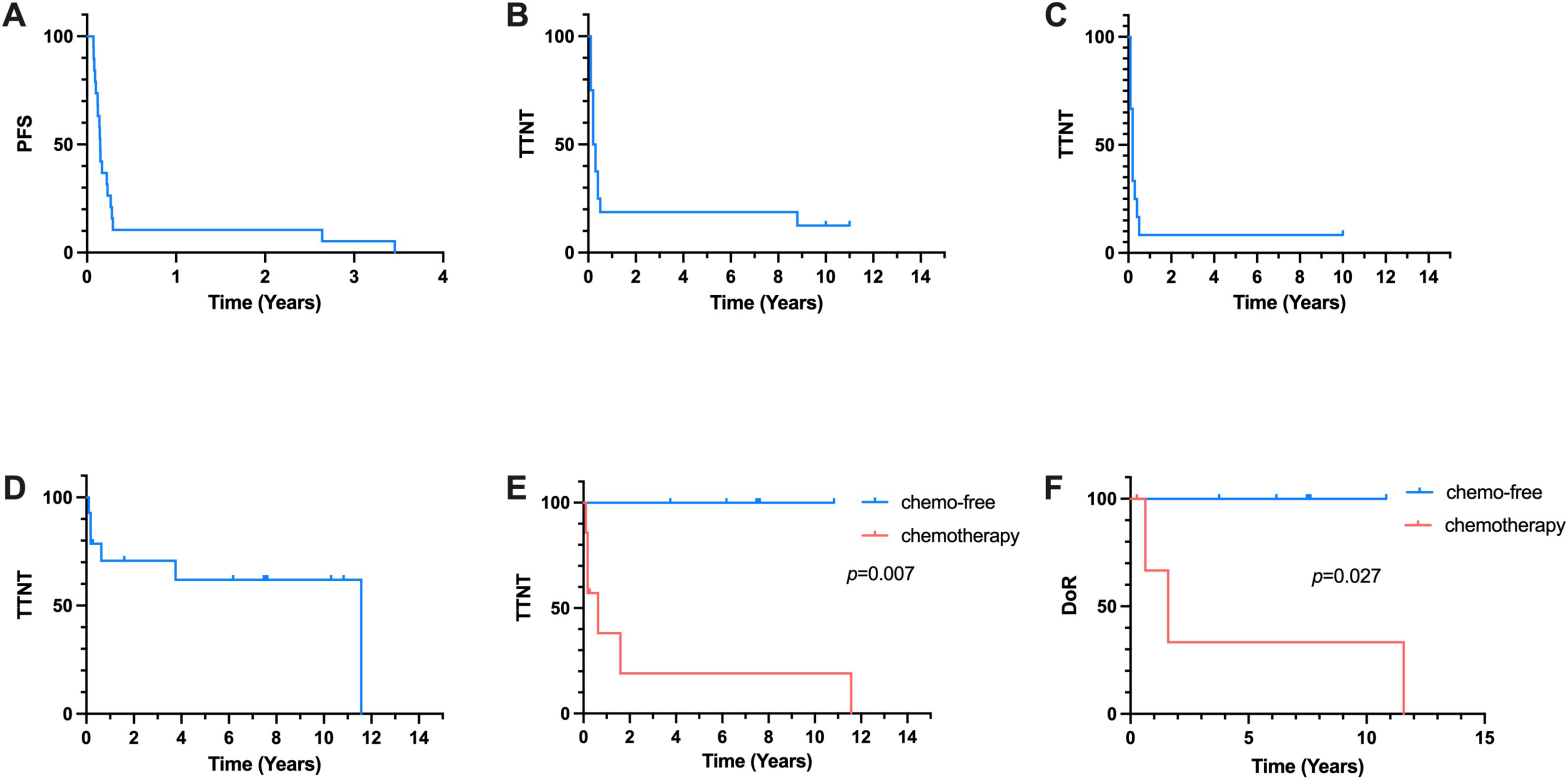
Kaplan-Meier Curve. **(A)** PFS of all 19 patients receiving first-line treatment. **(B)** TTNT of 16 patients reciving salvage second-line treatment. **(C)** TTNT of 12 patients accepted chemotherapy-based second-line treatment. **(D)** TTNT of all 14 patients received the third-line treatment. **(E)** TTNT of chemo-free group and chemotherapy group in the third-line treatment. **(F)** DoR of chemo-free group and chemotherapy group in the third-line treatment.

Twenty eight patients with newly-diagnosed SPTCL accepted chemotherapy-based treatment in our center. Sixteen primary refractory patients and three first relapsed patients of them were included between September 1997 and October 2020. Their clinical characteristics were summarized in **Table 1**. The median age was 34 (interquartile range(IQR), 14-48) years. 9 patients (47.4%) were male. 16 patients (84.2%) had B symptoms and 7 patients (36.8%) had concurrent hemophagocytic lymphohistiocytosis (HLH) according to HLH criteria 2004. In 8 patients who had extracutaneous sites involvement, 3 patients were found to have bone marrow involvement through bone marrow aspiration and biopsy. The other affected sites included lung, muscle and intestine. All patient received chemotherapy-based treatment as the first-line treatment and 15 patients (78.9%) received CHOP-like regimens. The cut-off date of last follow-up was April 2024, and the median follow-up time was 95.1 months (95% CI, 25.1 to 144.2 months). The median PFS of first-line treatment were 1.8 months (**Figure 1A**).

**Table 1:** Clinical characteristics of R/R SPTCL patients.

Characteristics	N(%)
Male	9 (47.4%)
Median Age, years	34 (range 14-48)
B symptom	16 (84.2%)
PIT: ≥2	11 (57.9%)
Extracutaneous site involved	8 (42.1%)
LDH (median, IQR) U/L	704, 219-947
Abnormal liver function	13 (68.4%)
HLH	7 (36.8%)
First-line chemotherapy	
CHOP	7 (36.8%)
CHOEP	8 (42.1%)
GDP-ML	4 (21.1%)
Response to initial chemotherapy	
Relapsed	3 (15.8%)
Refractory	16 (84.2%)
Median PFS (months)	1.8 (IQR 1.2-3.2)
Outcome	
Death	7 (36.8%)

PIT, prognostic index for T-cell lymphoma; HLH, Hemophagocytic Lymphohistiocytosis; CHOP, cyclophosphamide, hydroxydaunorubicin, vincristine and prednisone; CHOEP, cyclophosphamide, hydroxydaunorubicin, vincristine, etoposide and prednisone; GDP-ML, gemcitabine, dexamethasone, cisplatin, methotrexate, pegaspargase.

### Response to salvage therapy

16 patients received salvage therapies. The detailed treatment regimens and responses to treatment were listed as follows (**Table 2**). In the second-line treatment, the ORR was 31.3% and the median TTNT was 3.0 months (**Figure 1B**). Patient 1 and Patient 5 received allo-HSCT and achieved CR. The time to next treatment (TTNT) was 131.4 and 106.1 months. Patient 8 received cyclosproin A (CsA) plus interferon α (IFN α) regimen, achieved stable disease (SD) and a TTNT of 3.6 months later. Patient 16 achieved CR after accepting VRMP regimen but had a TTNT of 4.3 months later due to the patient’s discontinuation of medication on her own initiative. The other 12 patients accepted chemotherapy-based treatment. The ORR of these patients were 25% and the median TTNT was 2.4 months (**Figure 1C**). Seven of these twelve patients accepted regimen containing gemcitabine. The ORR was 28.6% and the relapse rate was 91.7%. Only Patient 13 who received bortezomib plus GDP (gemcitabin, cisplatin and dexamethasone) regimen achieved CR and has not relapsed for 120.3 months.

**Table 2:** Treatment regimens and responses of salvage treatment.

No	Age/sex	Initial therapy	Best Response	PFS, months	2nd therapy	Best Response	TTNT, months	3rd therapy	Best Response	TTNT, months	4th therapy	Best Response	TTNT, months	OS, months	Outcome
1	20/F	GDP-ML	PR	3.4	CHOP+HSCT	CR	131.4							134.8	Alive
2	32/M	CHOEP	PD	1.4	CsA+MTX+ MP	PD	1.5	FND	PD	2.2				4.9	Died
3	27/F	CHOEP	PD	1.7	Hyper-CVAD	PD	2.4	FND	CR	3.2				7.1	Alive
4	48/F	CHOP	PD	3.4	FND	CR	5.5	DICE	CR	138.8				147.5	Died
5	29/F	CHOP	PD	1.1	MOPP+ HSCT	CR	106.1	CHOP+HSCT	CR	123.7				230.1	Alive
6	45/M	GDP-ML	PD	2	MINE	PD	2.0	ESHAP	PD	1.3				4.6	Died
7	24/M	CHOP	SD	0.9	FND	PD	1.5	MINE	PD	2.1				4.3	Died
8	18/F	CHOP	PD	2.7	CsA+IFN	SD	3.6	VRMP	PR	74.1				80.2	Alive
9	36/M	CHOEP	PD	1.8	GDE+LEN	PD	1.5	MA	PR	7.6	CsA+INF	CR	11.2	22.1	Alive
10	26/M	CHOP	SD	3.5	GDE	SD	3.4	VRMP	CR	129.9				136.9	Alive
11	18/F	CHOEP	PD	1.5	GDE	PD	2.5	CsA+IFN	CR	91.4				95.1	Alive
12	14/M	CHOEP	PD	1.2	GDE	PD	1.2	VRMP	CR	89.6				91.7	Alive
13	35/F	GDP-ML	PD	3.2	GDP+Bort	CR	120.3							123.5	Alive
14	32/F	CHOEP+ chidamide	PD	1.8	GDE	PD	2.7	CsA+IFN	CR	90.6				95.1	Alive
15	34/M	CHOP+ chidamide	PR	32.1	GDP	CR	5.1	CsA+IFN	CR	45				82.2	Alive
16	22/F	CHOEP	PD	1.9	VRMP	CR	4.3	Azacitidine+ chidamide	PR	19.1	Tofacitinib+ CsA+chidamide	CR	15.6	42	Alive

CR, complete remission; PR, partial remission; SD, stable disease; PD, progressive disease; CHOP, cyclophosphamide, hydroxydaunorubicin, vincristine and prednisone; CHOEP, cyclophosphamide, hydroxydaunorubicin, vincristine, etoposide and prednisone; GDP-ML, gemcitabine, dexamethasone, cisplatin, methotrexate, pegaspargase; HSCT, Hematopoietic Stem Cell Transplantation; FND, fludarabine, mitoxantrone, dexamethasone; DICE, dexmethasone, ifosfamine, cisplatin, etoposide; Hyper-CVAD, megadose cyclophosphamide, doxorubicin, vincristine, and dexamethasone; MINE, mitoxantrone,ifosfamide, mesna and etoposide; MOPP, mechlorethamine, vincristine, procarbazine, prednisone; ESHAP, etoposide, methylprednisolone, cytarabine, cispalatin; VRMP, bortezomib, lenadomide and methylprednisolone; LEN, lenalidomide.

Fourteen patients received a third-line treatment. The ORR was 71.4% and the median TTNT was 138.8 months (**Figure 1D**). Seven patients accepted chemotherapy-based regimen and six patients accepted chemo-free regimen such as VRMP (bortezomib, lenadomide and methylprednisolone) regimen and CsA plus IFNα regimen. In chemotherapy group, all patients relapsed and 4 of them died of lymphoma progression. Patient 5 achieved CR and did not relapse until last follow-up. In chemo-free group, three patients received VRMP regimen treatment, all of whom had concurrent HLH. The other three patients received CsA plus IFNα regimen, one of whom had concurrent HLH. All patient in chemo-free group achieved CR or PR and did not relapse until last follow-up. The median TTNT of chemotherapy group was 7.6 months and the median TTNT of chemo-free group was not reached. Chemo-free group had a better TTNT than chemotherapy group (*p*=0.007) (**Figure 1E**). Among patients who achieved remission, the chemo-free group exhibited a longer DoR compared to the chemotherapy group (*p*=0.027) (**Figure 1F**). The chemo-free and chemotherapy regimens demonstrated similar safety profiles in terms of severe adverse events. The most common severe adverse events (AE) of chemo-free and chemotherapy regimens were both neutropenia (**Supplementary Table S1**).

Only Patient 9 and Patient 16 accepted the fourth line treatment. Patient 9 accepted CsA plus IFNα regimen and Patient 16 received tofacitinib, CsA plus chidamide regimen. Both of the regimens belonged to chemo-free therapy and they achieved CR.

### Overall survival

**Figure 2:**
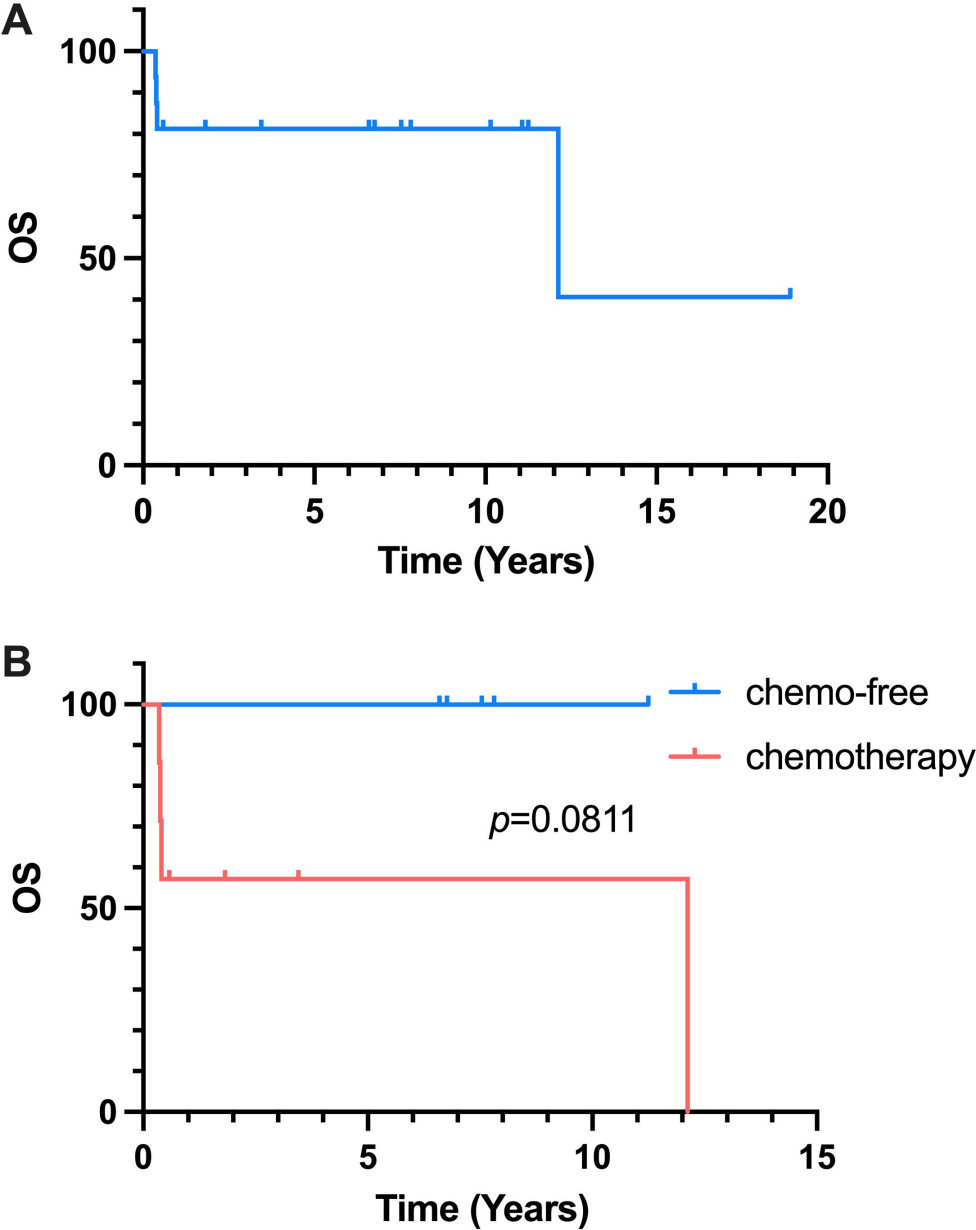
Kaplan-Meier Curve of OS. **(A)** OS of all 16 patients reciving salvage treatment. **(B)** OS of chemo-free group and chemotherapy group.

4 patients finally died in the follow-up owing to disease progression at the third-line chemotherapy treatment. The median OS and the OS rate of all patients receiving salvage treatment were 147.5 months and 75% (**Figure 2A**). After analyzing the chemotherapy and chemo-free groups among 14 patients receiving third-line treatment, the median OS were found to be 147.5 months and not reached in the chemotherapy and chemo-free groups, respectively (**Figure 2B**). The estimated 1-year OS rates of two groups were 57.1% vs 100%.

### Outcome analysis

We conducted the Cox proportional hazards regression analysis to test which prognostic factors could predict the TTNT of the second-line and third-line treatment in R/R SPTCL patients. The results of univariate analysis of outcome were summarized in **Supplementary Table S2**. Sex (*p*=0.027) was found to significantly affect TTNT of the second-line in univariate analysis. And the using of chemo-free regimen in the third-line was also found to affect TTNT of the third-line treatment significantly in univariate analysis. Previously we reported that HLH was indicator of shorter PFS of first-line treatment in patients with newly-diagnosed SPTCL in univariate analysis. However, in this study, HLH didn’t significantly affect the outcome in patients with R/R SPTCL. Seven patients with concurrent HLH received the third-line treatment, of whom 3 accepted VRMP regimen, 3 accepted chemotherapy-based regimen and 1 accepted CsA plus IFNα. All patients receiving chemo-free regimens achieved CR or PR and did not relapse until last follow-up. No significant predictor of the OS was found.

## Discussion

Due to the rarity of SPTCL, most previous studies were retrospective analyses or case reports, and no retrospective studies of R/R SPTCL have been reported. Thus, investigating the clinical characteristics and treatment outcomes in Chinese R/R SPTCL patients is essential.

This single-center, retrospective study examines the clinical profiles and salvage treatments of 19 patients with R/R SPTCL. To our knowledge, this represents the first and largest reported series of R/R SPTCL ever reported. Our study highlighted that chemo-free immunomodulatory salvage regimens such as CsA plus INFα and VRMP, may be superior to traditional cytotoxic chemotherapy regimens.

A previous literature has compared two different first-line treatment of SPTCL: immunomodulatory therapy and traditional multi-drug chemotherapy. Immunomodulatory therapy appeared to demonstrate better therapeutic outcomes (4, 8, 11). In this study, in 28 patients accepted chemotherapy-based treatment as the first-line treatment, 19 patients experienced early relapse or were refractory, which indicated that chemotherapy as a first-line treatment was ineffective. Recent studies also reported that immunomodulatory drug such as histone deacetylase inhibitor (13) and lenalidomide (14) showed excellent effect in SPTCL. Recurrent mutations in epigenetic modifiers and the PI3K/AKT/mTOR pathway were also reported in SPTCL, which also suggested the potential mechanism of action of immunomodulatory drugs in SPTCL (15).

In our study, among the 14 patients with R/R SPTCL who received third-line treatment, 6 patients received chemo-free immunomodulatory treatment, including CsA plus INFα regimen and VRMP regimen, as salvage regimens. The median TTNT of chemotherapy group was 7.6 months and the median TTNT of chemo-free group was not reached. This result showed that in third-line treatment, the chemo-free group had better disease control rates and TTNT than chemotherapy group (*p*=0.007). However, there are currently no other prospective researches on salvage therapy for R/R SPTCL to help us analyze the efficacy of the chemo-free and chemotherapy regimens. Moreover, two patients achieved HSCT as second and third line treatment also achieved long-term remission more than 100 months.

In this study, cyclosporine A demonstrated efficacy in the patients with R/R SPTCL. Cyclosporine A is believed to inhibit the function of lymphocytes by reducing the production of inflammatory cytokines from T-lymphocytes. However, the precise mechanism by which cyclosporine A treats SPTCLs remains unclear. Chen et al. reported that a relapsed SPTCL patient achieved a complete and sustained response to oral cyclosporine A as a single agent after failing salvage chemotherapy and high-dose dexamethasone (16). In relapsed SPTCL patients with HLH, high-dose steroids and cyclosporine A have been reported to effectively control symptoms (17). The response of cyclosporine A was durable in most cases, and it was reported that seven patients even sustained remission after discontinuing cyclosporine A treatment (16). Furthermore, the patients may benefit from cyclosporin A retreatment (18, 19). Cyclosporin A also demonstrated efficiency in patients with visceral involvement (19–21). In our series, 3 SPTCL patients without hyperinflammation were treated with cyclosporine A as a third-line therapy. All 3 patients achieved durable and sustained remission. Our findings and review showed that cyclosporine A has a significant anti-lymphoma effect on SPTCL. These encouraging findings suggest that cyclosporine A could serve as a cornerstone treatment for R/R SPTCL. However, the optimal dosage, schedule of CsA and potential adjuvant for CsA agents are not determined. More investigations are needed to clarify the way cyclosporine A functions in SPTCLs.

The VRMP regimen was developed based on the assumption that HD-MP can rapidly control the severe inflammation. Constitutive activation of the transcription factor nuclear factor kappa B (NFκB) has been confirmed in CTCL, with NF-kB activation associated with resistance to cell death, potentially explaining treatment failure. Bortezomib, a proteasome inhibitor, can suppress the NF-κB activation and induce cell apoptosis. Bortezomib has been widely used in multiple myeloma and active-B-cell origin Diffuse large B cell lymphoma (22, 23). Thus, we hypothesized that a bortezomib-based regimen could be effective in R/R SPTCL. Lenalidomide is an orally active immunomodulatory drug with both direct antineoplastic activity and indirect effects on tumor cells and tumor microenvironment. It has been reported that single-agent use can display durable responses in R/R NHL (24, 25). In our study, 3 patients with concurrent HLH were treated with VRMP regimen as a third-line therapy, achieving an ORR rate of 100%. All of them achieved persistent complete remission. This result reveals the efficacy of VRMP regimen.

Despite the fact that the 5-year overall survival rates were reported to be 91% and 46% in SPTCL patients with and without HLH, the data is unknown for R/R patients before (26). Our results showed that of 16 R/R SPTCL patients who received salvage therapies, 4 patients died. The OS rate among these patients was 75%. Additionally, the estimated 1-year OS rates was 57.1% in chemotherapy group and 100% in chemo-free group. Most patients receiving chemo-free treatment had accepted more than one line of previous polychemotherapy. These patients had undergone extensive cytotoxic treatment, complicating their disease status and worsening their prognosis. Despite this, the chemo-free group demonstrated surprisingly positive responses, superior outcomes, and a one-year OS rate of 100%, underscoring the advantages of chemo-free regimens over chemotherapy.

Our study also had limitations. First, our data were derived from retrospective and observational studies, which could be influenced by various factors. Second, due to the rarity of SPTCL disease itself, our result was limited by its small population. Moreover, we did not analyze the mutational landscape of SPTCL to explore the relationship between mutations and prognosis. More and larger-scale studies were needed to validate the efficacy of chemo-free immunomodulatory regimen in R/R SPTCL.

This study represents the largest single-center retrospective cohort of R/R SPTCL. For patients who fell in the first-line conventional polychemotherapy, chemo-free immunomodulatory regimen may be a better choice compared with cytotoxic chemotherapy. CsA and bortezomib-based regimen exhibited excellent efficacy and better outcome. This study had a long follow-up period, demonstrating the durability of immunomodulatory drugs. Despite the promising clinical responses, further trials are warranted to validate our conclusions.

## Data Availability

The original contributions presented in the study are included in the article/**Supplementary Material**. Further inquiries can be directed to the corresponding author.

## Supplementary material

Supplementary Table 1 Safety profile of chemo-free group and chemotherapy group in third-line treatment.

Supplementary Table 2 Univariate Cox regression analysis of the TTNT of the second-line and TTNT of third-line treatment and the OS.
