# Supplementary material for: Chemo-free salvage treatment outperforms traditional chemotherapy in advanced lines of relapsed/refractory subcutaneous panniculitis-like T-cell lymphoma
Source: Front Immunol. 2024 Dec 9;15:1476875. doi: 10.3389/fimmu.2024.1476875 (PMC11663913; doi:10.3389/fimmu.2024.1476875)
Supplement: Supplementary Table 1 — Safety profile of chemo-free group and chemotherapy group in third-line treatment. [file Table1.docx]

|  | chemo-free group, n (%) | chemotherapy group, n (%) |
| --- | --- | --- |
| Severe AEs (Grade 3 or 4) |  |  |
| Neutropenia | 2, 33.3% | 2, 28.6% |
| Anemia | 1, 16.7% | 1, 14.3% |
| Thrombocytopenia | 1, 16.7% | 1, 14.3% |
| Infective AEs | 1, 16.7% | 1, 14.3% |
| Nausea and vomiting | 1, 16.7% | 0 |
| Infusion reaction | 0 | 1, 14.3% |
| Hepatobiliary disorder | 0 | 1, 14.3% |
| Renal damage | 0 | 1, 14.3% |

Table S1. Safety profile of chemo-free group and chemotherapy group in third-line treatment.

| Variables | | TTNT of second-line treatment | | TTNT of third-line treatment | |
| --- | --- | --- | --- | --- | --- |
|  |  | HR (95% CI) | *p* value | HR (95% CI) | *p* value |
| Sex | Male | 3.526(1.083-11.475) | 0.027 | 3.444(0.661-17.935) | 0.142 |
| Age | Years | 0.992(0.936-1.051) | 0.779 | 1.013(0.939-1.092) | 0.747 |
| B symptom | yes | 1.074(0.293-3.934) | 0.914 | 38.499(0.039-37786.803) | 0.299 |
| Hepato/  splenomegaly | yes | 1.222 (0.415-3.598) | 0.716 | 2.173(0.418-11.294) | 0.356 |
| WBC | ×10^9^ | 1.065(0.957-1.185) | 0.249 | 1.052(0.923-1.199) | 0.447 |
| TNM staging | ≥III | 1.569(0.531-4.631) | 0.412 | 6.026 (0.723-50.227) | 0.097 |
| PIT | ≥2 | 0.805(0.281-2.306) | 0.686 | 7.524 (0.898-63.048) | 0.063 |
| LDH | U/L | 1.001(1.000-1.002) | 0.172 | 1.002(1.000-1.003) | 0.074 |
| Ab. Liver function | yes | 0.776(0.253-2.379) | 0.658 | 4.204(0.503-35.098) | 0.185 |
| ESR | mm/h | 0.975(0.941-1.010) | 0.163 | 1.030(0.989-1.074) | 0.157 |
| HLH | yes | 2.435(0.748-7.932) | 0.140 | 1.232(0.275-5.512) | 0.785 |
| Chemo-free regimen in third-line | yes | / | / | 0.114(0.013-0.972) | 0.047 |

Table S2 Univariate Cox regression analysis of the TTNT of the second-line and TTNT of third-line treatment and the OS.
